# Evaluation of bi-directional causal association between periodontitis and benign prostatic hyperplasia: epidemiological studies and two-sample mendelian randomization analysis

**DOI:** 10.3389/fgene.2024.1326434

**Published:** 2024-04-10

**Authors:** Haotian Wei, Guangjie Tian, Shendan Xu, Yaqi Du, Minting Li, Yonglan Wang, Jiayin Deng, Changyi Quan

**Affiliations:** ^1^ Department of Urology, The Second Hospital of Tianjin Medical University, Tianjin, China; ^2^ Periodontal Department, School and Hospital of Stomatology, Tianjin Medical University, Tianjin, China

**Keywords:** periodontitis, prostatic hyperplasia, mendelian randomization analysis, epidemiologic studies, genetic association studies

## Abstract

**Background:** Periodontitis and benign prostatic hyperplasia (BPH) are all common chronic diseases with higher incidence in middle-aged and old men. Several studies have indicated a potential association between periodontitis and BPH, although the findings remain inconclusive. However, there is no mendelian randomization (MR) studies to assess this association.

**Methods:** The 40 men who had received health check-ups were included in an epidemiological study. Genetic data of BPH (13118 cases and 72799 controls) and periodontitis (3046 cases and 195395 controls) from FinnGen project was used to perform two-sample MR analysis. The inverse-variance weighted (IVW) model was identified as the primary analytical method, with MR Egger, weighted median, simple mode, and weighted mode serving as additional approaches.

**Results:** The epidemiological analysis demonstrated a lack of statistically significant differences in the prevalence of clinical BPH between severe periodontitis group and non-severe periodontitis group. Similarly, no statistically significant differences were found in the prevalence of severe periodontitis among individuals with clinical BPH compared to those without. Additionally, Among the five models utilized in MR analysis, including the IVW model, no evidence of a causal link between periodontitis and BPH was observed.

**Conclusion:** The findings from our epidemiological investigation and MR analysis do not provide support for a causal relationship between periodontitis and BPH.

## Introduction

Periodontitis is a chronic inflammatory disease caused by plaque biofilm and multiple factors, resulting in destruction of the periodontal supportive tissues (Page et al., 1997; Slots, 2017). Severe periodontitis may affect approximately 11% of the global population, thereby impacting 743 million individuals (Kassebaum et al., 2014; Richards, 2014; Frencken et al., 2017). The 4th National Oral Health Survey in China reveals a significant prevalence of periodontitis among Chinese adults, with rates of 52.8%, 69.3%, and 64.6% observed in the age groups of 35–44, 55–64, and 65–74 years, respectively (Jiao et al., 2021). This considerable prevalence imposes a significant burden on individuals, households, and society at large. As a chronic inflammatory disease, the causative factors of periodontitis are complex (Loos and Van Dyke, 2020). Various genetic and environmental factors are known to interact in the pathogenesis of periodontitis, with alterations in the periodontal microbial community playing a significant role (Papapanou et al., 1997; Socransky et al., 1998; Ebersole et al., 2016). The presence of periodontal pathogenic bacteria within the gingival tissues is proposed to be a crucial factor in the pathogenesis of periodontitis (Ji et al., 2015). Periodontal bacteria typically maintain a symbiotic relationship with the host depending on the protective actions of neutrophils and antimicrobial peptides (Dixon et al., 2004; Dale and Fredericks, 2005). Periodontal pathogenic bacteria exhibit notable resistance to immune defenses, resulting in a disruption of homeostasis between the host and the periodontal microbial community (Ji et al., 2007a; Ji et al., 2007b; Hajishengallis et al., 2011). The continual presence of periodontal pathogens results in the mobilization of escalating quantities of inflammatory cells within the gingival tissues, culminating in tissue destruction. Various immune cells, such as neutrophils and helper T cells, are pivotal in this process (Kantarci et al., 2003; Bunte and Beikler, 2019). While periodontal pathogenic bacteria serve as the primary etiological factor of periodontitis, genetic predisposition, lifestyle choices, and systemic diseases also contribute to the development of periodontitis. Genetic variations have the potential to induce modifications in the immune response, thereby influencing an individual’s vulnerability to periodontitis (Michalowicz et al., 2000; Vaithilingam et al., 2014). Additionally, lifestyle factors such as smoking and other chronic inflammatory conditions may alter the course of periodontitis by impacting immune function (Reynolds, 2014; Bunte and Beikler, 2019). Consequently, the effective prevention and treatment of periodontitis necessitate prioritizing the reduction of modifiable risk factors. In addition to removing oral local promoting factors, systemic promoting factors cannot be ignored (Kwon et al., 2021). Furthermore, periodontitis has been found to have negative implications on various non-communicable chronic diseases, such as benign prostatic hyperplasia (BPH) (Wu et al., 2019), metabolic syndrome (Aizenbud et al., 2023), and chronic obstructive pulmonary disease (Xiong et al., 2023).

BPH, a prevalent chronic disease, is characterized by the excessive growth of smooth muscle and epithelial cells in the prostatic transition zone (Devlin et al., 2021). Patients diagnosed with BPH may present with symptoms including increased urinary frequency, urgency, and dysuria (Speakman et al., 2015). One study has revealed that until 2010, over 210 million men suffered from BPH (Vos et al., 2012). Histologic diagnosis has traditionally served as the benchmark for diagnosing BPH; however, not all patients exhibit distressing symptoms (Foo, 2017). Consequently, the diagnosis of clinical BPH holds greater significance for the patient. The evaluation of obstructive symptoms can be initially determined through the assessment of prostate volume and the International Prostate Symptom Score (IPSS) (Foo, 2019). In addition, uroflowmetry is also a crucial diagnostic indicator in assessing BPH (Pandolfo et al., 2023). The etiology of BPH is multifactorial, with aging and hormonal imbalances being widely acknowledged as primary contributors (Devlin et al., 2021). Additionally, genetic predisposition, chronic inflammation, and metabolic alterations have been identified as potential factors influencing the progression of BPH. Specific genetic risk factors associated with BPH include Y chromosome deletions and certain single nucleotide polymorphisms (SNP) (Aly et al., 1994; Salam et al., 2005). Nevertheless, chronic inflammation results in alterations in inflammatory cell infiltration and associated growth factors, which not only trigger BPH but also expedite its advancement (Giri and Ittmann, 2000; Ficarra et al., 2014). Recent research suggests that periodontitis may serve as a potential risk factor for BPH (Boland et al., 2013; Wu et al., 2019; Hyun et al., 2021). In conjunction with prior research, we have discovered an intriguing association between periodontitis and BPH.

However, the available evidence regarding the causal association between periodontitis and BPH remains constrained, as only observational studies have been conducted. In recent years, Mendelian randomization (MR) has gained popularity as a novel analytical approach in accessing causality between exposure phenotype and outcome phenotype (Emdin et al., 2017). By employing genetic variation as instrumental variables (IVs), MR provides a better assessment of the causal relationship between exposure and outcome (Lawlor et al., 2008). Since the alleles of the genetic variant are assigned to each individual without any prior exposure, this results in its irrelevance to potential confounders (Evans and Davey Smith, 2015). Therefore, this methodology is particularly advantageous in mitigating bias stemming from confounding variables and reverse causality in observational research, offering a practical and efficient alternative in situations where randomized controlled trials are impractical (Taylor et al., 2014; Sekula et al., 2016). However, MR studies have not yielded any evidence to support the claim of a causal relationship. Hence, the objective of this study is to explore whether there exists a bidirectional causal relationship between periodontitis and BPH with MR.

## Materials and methods

### Observational epidemiological analysis

A cross sectional study was used. From September 2021 to September 2023, we collected patient demographics and examination data who in their 40 s or 50 s received periodontitis health check-up in Stomatological Hospital of Tianjin Medical University. Periodontitis, a chronic inflammatory disease with multifactorial origins, is characterized by clinical attachment loss (CAL), radiographically evaluated alveolar bone loss, the presence of periodontal pocketing, and gingival bleeding, as outlined by the clinical criteria established by the American Academy of Periodontology and the European Federation of Periodontology (Papapanou et al., 2018). Given the high prevalence of periodontitis in Chinese adults (62.3%) and the fact that severe periodontitis (stage III or IV) possesses higher clinical significance, our study focused primarily on severe periodontitis (Jiao et al., 2021). As not all individuals with BPH experience distressing symptoms, we selected clinical BPH as the primary focus of our study (Foo, 2017). Transabdominal ultrasonography and the IPSS were utilized to evaluate clinical BPH. The IPSS score less than or equal to 7 is mild, 8–19 represents moderate symptoms, and 20–35 represents severe symptoms. Significance testing by Fisher’s exact test or Wilcoxon rank-sum test as appropriate.

### Study participant criteria

Patients will be sifted according to the inclusion/exclusion criteria. Subjects eligible to participate in this study should meet all the following criteria: 1) age ≥40 years and ≤60 years, 2) male, and 3) Received both the periodontitis health check-up and transabdominal ultrasonography within a short period of time.

Based on previous literature report, age and diabetes mellitus are all shared risk factors for BPH and periodontitis (Parsons, 2007; Genco and Borgnakke, 2013). The exclusion criteria were as follows: 1) diabetes mellitus, 2) pyuria, and 3) suffering from any serious medical conditions.

### Diagnostic criteria

Severe periodontitis was defined as a 30% increase in the number of probed sites with a CAL of ≥5 mm among all probed sites (Papapanou et al., 2018). Using a Williams periodontal probe, with a force of 20–25 g, inserted parallel to the vertical axis of the tooth. The probing depth (PD) is the distance to which a probe penetrates into the pocket (Hassell et al., 1973). PD was measured by distal, central, and mesial on the buccal and lingual surfaces, and six positions were recorded for each tooth tested. The CAL is determined by subtracting from the depth of the pocket the distance from the gingival margin to the cemento-enamel junction (CEJ). When the gingival margin is located on the anatomic crown, the CAL is determined by subtracting from the depth of the pocket the distance from the gingival margin to the CEJ. If the two numbers are subtracted to zero or the CEJ cannot be probed, there is no CAL. When the gingival margin is located apical to the CEJ, the CAL is determined by adding from the depth of the pocket the distance from the gingival margin to the CEJ. CAL also recorded six positions for each of the tested teeth (Sivertson and Burgett, 1976).

Clinical BPH was defined as that seen in men showing moderate to severe symptoms (IPSS score ≥8 points) with prostate volume (PV)≥40 mL (Barry et al., 2017; Foo, 2019). PV was obtained using the prostate ellipsoid formula: $PV=\frac{\pi }{6}\times \text{height }\left(\text{cm}\right)\times \text{width }\left(\text{cm}\right)\times \text{length}\left(\text{cm}\right)$ . IPSS is a widely employed questionnaire for assessing BPH severity (Wong et al., 2017). Originally created by the American Urological Association, the IPSS questionnaire has demonstrated reliability and validity in research settings (Barry et al., 1992).

### Sample size estimation

In this study, the minimum sample size was determined utilizing the “Tests for Two Proportions” function within the PASS software. According to the results presented by Hana et al., the prevalence of BPH was reported as 5.3% in the non-periodontitis group and 21.4% in the periodontitis group (Hyun et al., 2021). Given the more stringent diagnostic criteria utilized for clinical BPH in our study, the estimated prevalence of BPH was found to be 3% in the non-periodontitis group and 20% in the periodontitis group. Employing a power of 80% and an α level of 0.1 for a one-sided test, the calculated sample size required for the study is 60. As a result of the rigorous inclusion criteria and challenges in collecting cases, 40 patients were enrolled in this study with 20 patients diagnosed with severe periodontitis. All participants were yellow race. The mean age of all participants was 49 years.

### Data sources in mendelian randomization

The GWAS data for periodontitis and BPH were extracted from FinnGen project (https://www.finngen.fi/en). The FinnGen study is a continuing research endeavor that leverages samples from a comprehensive network of Finnish biobanks and digital healthcare information from national health registries. The primary objective of FinnGen is to generate genomic data linked to health registry information for 500,000 biobank participants. As a substantial biobank resource characterized by the unique attributes of the Nordic healthcare system and population composition, FinnGen offers significant potential for diverse genetic investigations (Kurki et al., 2023). The DF9 release (May 2023) of the FinnGen consortium data was used, which contains 3046 cases and 195395 controls for periodontitis and 13118 cases and 72799 controls for BPH.

### Statistical analysis for mendelian randomization

**FIGURE 1:**
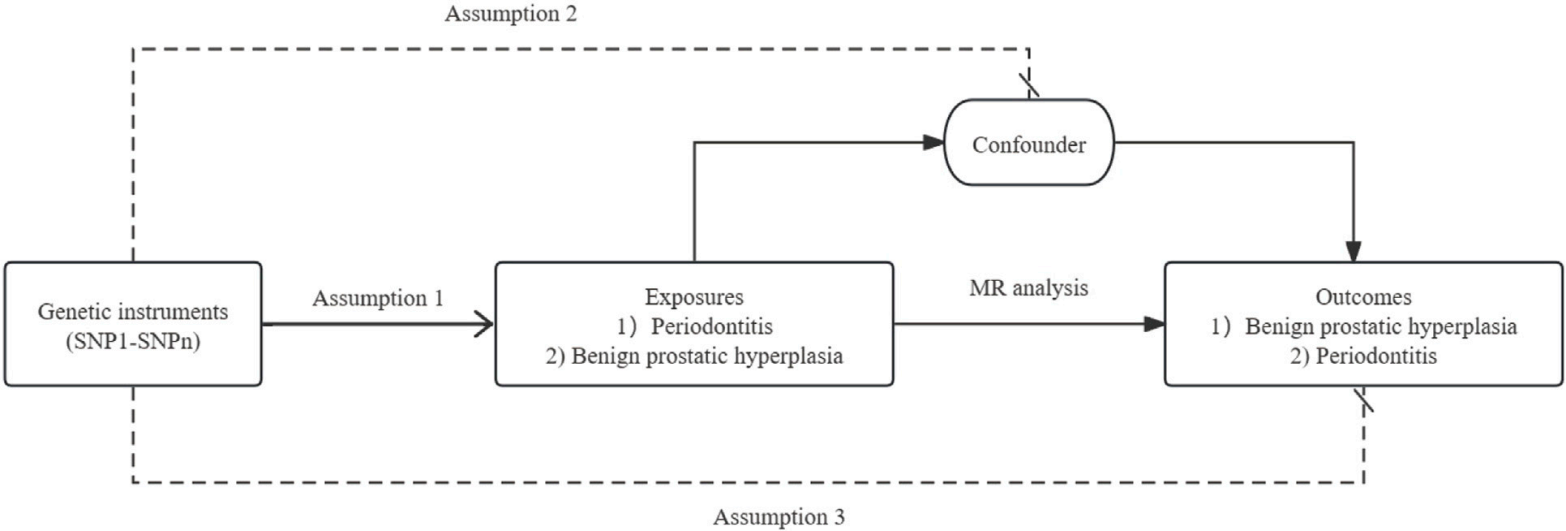
The schematic of the MR analysis of periodontitis and BPH.

This study employed a two-sample MR design to investigate the reciprocal causal relationships between periodontitis and BPH. Figure1 shows a schematic of the MR analysis of periodontitis and BPH. The study adhered rigorously to the three core assumptions of MR analysis: 1) The IVs exhibit a strong association with the exposure variable; 2) The IVs solely influence the outcome through their impact on the exposure; and 3) The IVs are presumed to have no correlation with any confounding factors influencing the exposure-outcome association (Davey et al., 2020). All datasets utilized in this investigation are publicly accessible, and ethical approval and written informed consent were obtained from the original studies.

In adherence to the three essential steps in the MR analysis process, rigorous quality control measures were implemented to ascertain the strength and dependability of the MR analysis. Initially, SNPs linked to the exposure variable were identified (*p* < 1 × 10^−6^). Subsequently, SNPs displaying significant linkage disequilibrium (LD) were removed to prevent potential biases in the results (r2 < 0.005, clumping distance = 2,000 kb). Lastly, SNPs associated with the outcome variable were excluded from the analysis (*p* < 1 × 10^−5^). F-statistics were computed to evaluate the efficacy of each IV in our study (Hartwig et al., 2017). The use of F-statistics is a common practice in statistical analysis for assessing the strength of instrumental variables. While IV techniques are generally unbiased in the presence of confounding factors, estimates derived from IV analysis can be subject to finite sample bias, commonly referred to as weak instrument bias. This bias tends to align with the observed confounded relationship and its extent is influenced by the strength of the association between the genetic instrument and the phenotype. The F- statistics is capable of detecting and quantifying this bias; a higher F-value indicates a lesser degree of bias (Burgess et al., 2011).

*R* ^2^ was calculated as follows: $R^{2}=2\times \left(1-EAF\right)\times EAF\times {\left(\frac{BETA}{SE\times \sqrt{N}}\right)}^{2}$

F-statistics was calculated as follows: $F=\frac{N-k-1}{k}\times \frac{R^{2}}{1-R^{2}}$

The inverse-variance weighted (IVW), MR-Egger, weighted median, weighted mode, and simple model were used to examine a causal association (Burgess et al., 2013). The IVW model serves as the primary foundation for our conclusions, with the other four models utilized as supplementary frameworks. The IVW model, a robust weighted linear model, necessitates the validity of all genetic variants as instrumental variables (Hartwig et al., 2017). In contrast, the remaining four models offer more flexibility in terms of instrumental variable requirements. The MR-Egger regression method is capable of producing unbiased estimates in the presence of pleiotropy within instrumental variables (Bowden et al., 2015). Weighted medians, with a minimum weight of 50%, offer robust estimates of influential instrumental variables (Bowden et al., 2016). The weighted mode is effective when a majority of instrumental variables are deemed valid (Li et al., 2022). The simple mode, as a model-based assessment strategy, provides resilience against pleiotropy (Hartwig et al., 2017; Yang et al., 2023)

The MR-Egger tested its horizontal pleiotropic (Hemani et al., 2018a). The IVW and MR-Egger were used to quantify the heterogeneity effect between the genetic instruments (Bowden et al., 2015; Bowden et al., 2016). Finally, we tested the consistency of the results by leave-one-out analysis. Data analysis in this study was performed using R (version4.2.1) through TwoSampleMR (0.5.6) package and MRPRESSO (1.0) package (Hemani et al., 2018b).

## Results

### Observational epidemiological analysis

A total of 40 male patients, with an average age of 49 ± 4.84 years old, were enrolled in our study. Among these patients, 20 had severe periodontitis and 8 had clinical BPH. Our analysis revealed no statistically significant differences in PV (*p* = 0.82), IPSS (*p* = 0.799), and the prevalence of clinical BPH (*p* = 0.454) between severe periodontitis group and non-severe periodontitis group. Similarly, no statistically significant differences were observed in CAL (*p* = 0.778) and the severe prevalence of severe periodontitis (*p* = 0.695) between clinical BPH group and non-clinical BPH group. Further details regarding the characteristics of the participants can be found in Table 1.

**TABLE 1:** Baseline characteristics of the participants (*n* = 40).

Variable	Total (*n* = 40)	Severe periodontitis (+)	Severe periodontitis (−)	*p*-value	Clinical BPH (+)	Clinical BPH (−)	*p*-value
**Age (year)**	49 ± 4.84	48.4 ± 4.24	49.6 ± 5.41	0.414	50.25 ± 4.89	48.69 ± 4.85	0.517
**Prostate volume (mL)**	35.02 ± 12.88	35.51 ± 14.84	34.54 ± 10.95	0.820	53.45 ± 11.25	30.42 ± 8.4	0.000
**IPSS score**	4.08 ± 3.32	3.95 ± 3.43	4.2 ± 3.32	0.799	9.13 ± 1.46	2.81 ± 2.28	0.000
**CAL (mm)**	3.54 ± 1.23	4.40 ± 1.01	2.69 ± 0.74	0.000	3.52 ± 0.80	3.55 ± 1.33	0.829
**Severe Periodontitis**	20 (50%)	NA	NA		5 (62.5%)	15 (46.9%)	0.695
**Clinical BPH**	8 (20%)	3 (15%)	5 (25%)	0.454	NA	NA	NA

### Mendelian randomization

The cohorts utilized in the MR analysis to obtain the genetic instruments consisted of 3,046 patients diagnosed with periodontitis (with 195,395 controls) and a total of 13,118 patients diagnosed with BPH (with 72,799 controls). After considering a *p*-value threshold of less than 5 × 10^−6^ and chain imbalance, as well as excluding relevant SNPs, a total of 17 SNPs were selected as genetic instruments (Supplementary Table S1). The F-statistic for these SNPs ranged from 10.2 to 26.2, indicating their robustness as IVs.

**FIGURE 2:**
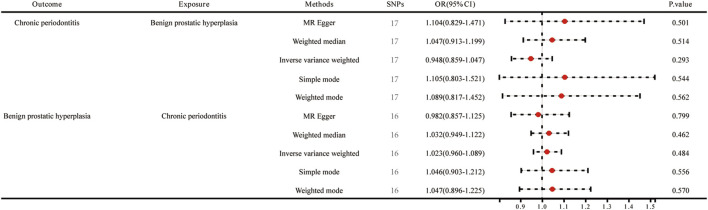
Forest plots of MR estimates for the relationship between genetically instrumented BPH and periodontitis.

**FIGURE 3:**
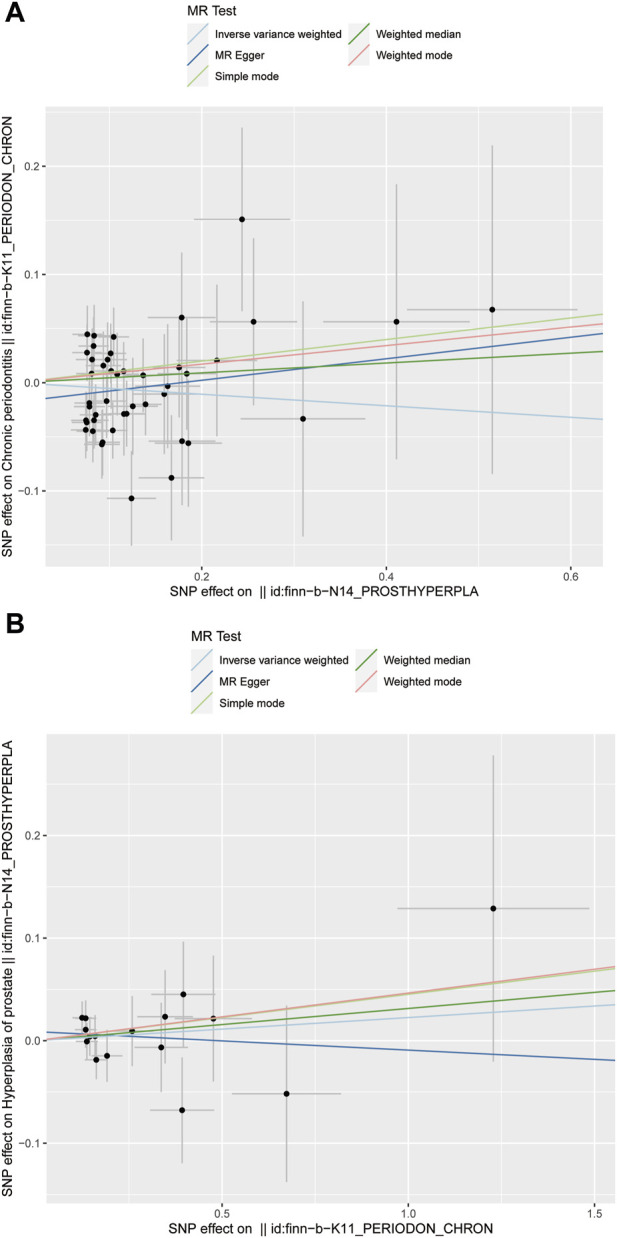
Scatter plot of the causal relationships between benign prostatic hyperplasia and periodontitis using different MR methods. **(A)** Causal estimates for BPH on periodontitis; **(B)** Causal estimates for periodontitis on BPH.

**FIGURE 4:**
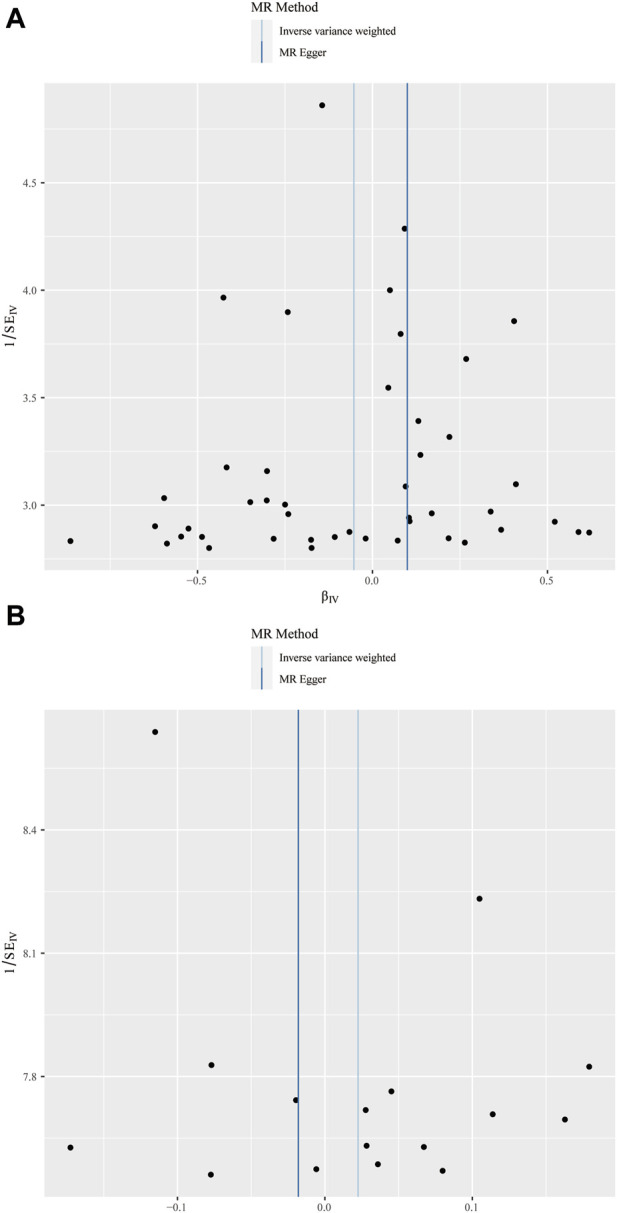
Funnel plot of the causal relationships between benign prostatic hyperplasia and periodontitis. **(A)** MR estimates for BPH on periodontitis; **(B)** MR estimates for periodontitis on BPH. The funnel plot illustrated the overall symmetry of causal estimates across all instrumental variables.

**FIGURE 5:**
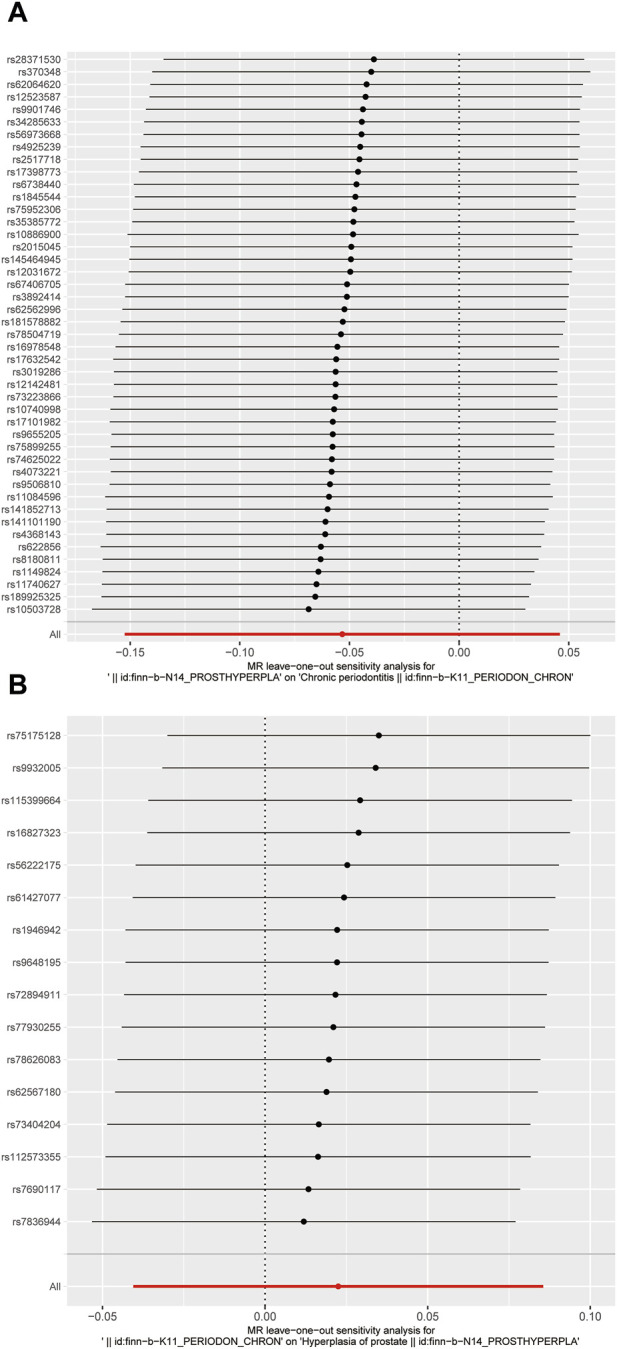
Forrest plot of the causal relationships between benign prostatic hyperplasia and periodontitis. **(A)** MR estimates for BPH on periodontitis; **(B)** MR estimates for periodontitis on BPH.

In this study, various MR methods were employed to investigate the potential causal relationship between BPH and periodontitis. The findings revealed no significant evidence supporting a causal effect of BPH on periodontitis, as indicated by the IVW analysis (OR = 0.948, *p* = 0.293) (Figure 2; Figure 3A). Furthermore, comprehensive multiplicity and sensitivity analyses were conducted to assess the robustness of this causal relationship. The results of the MR-Egger intercept test (Intercept = −0.018, *p* = 0.273) (Table 2) indicated no evidence of directional horizontal pleiotropy, suggesting that the SNPs associated with BPH did not influence the incidence of periodontitis through mechanisms unrelated to BPH. No asymmetry was noted in the funnel plot, indicating that directional horizontal pleiotropy was not detected (Figure 4A). The *p*-values of MR-Egger and IVW methods were 0.206 and 0.197, respectively, providing a scanty demonstration of heterogeneity among the SNPs. According to the leave-one-out analysis (Figure 5A), no significant SNPs drove the causal relationship between BPH and periodontitis. In the reverse MR analysis, we found no significant results sustaining the latent reverse causation between BPH and periodontitis with 16 periodontitis related SNPs (Figure 2; Figure 3B). And the heterogeneity and pleiotropy tested negative (Figure 4B; Figure 5B). And all the abbreviations used in this paper are summarized in Table 3.

**TABLE 2:** Pleiotropy tests and heterogeneity of MR.

BPH-PD								
Pleiotropy test			Heterogeneity test					
			MR egger			IVW		
Egger_intercept	SE	*p*-value	*Q*	*df*	*Q*_*p*-value	*Q*	*df*	*Q*_*p*-value
−0.018	0.016	0.273	50.32	43	0.2061	51.77	44	0.1965
PD-BPH								
**Pleiotropy test**			**Heterogeneity test**					
			**MR egger**			**IVW**		
Egger_intercept	SE	*p*-value	*Q*	*df*	*Q*_*p*-value	*Q*	*df*	*Q*_*p*-value
0.0088	0.013	0.521	8.499	14	0.8617	8.931	15	0.8811

**TABLE 3:** The list of abbreviations.

Abbreviations	Unabbreviated form
BPH	benign prostatic hyperplasia
IPSS	International Prostate Symptom Score
SNP	Single nucleotide polymorphism
MR	Mendelian randomization
IVs	Instrumental variables
CAL	Clinical attachment loss
PD	Probing depth
CEJ	Cemento-enamel junction
PV	Prostate volume
LD	Linkage disequilibrium
IVW	Inverse-variance weighted

## Discussion

This study undertook an assessment of the causal association between periodontitis and BPH through the utilization of both observational analysis and MR analysis. The findings of our study indicate the absence of a substantial causal relationship between periodontitis and BPH.

Initially, we obtained health check-up data from a cohort of 20 individuals diagnosed with severe periodontitis and another 20 individuals diagnosed with non-severe periodontitis. To avoid the effects of potential shared risk factors between periodontitis and BPH, we established rigorous exclusion criteria, which encompassed diabetes mellitus, pyuria, and the presence of any significant medical conditions. Subsequently, we evaluated the occurrence of clinical BPH in the aforementioned 40 patients through the utilization of PV and IPSS assessments. In the group with severe periodontitis, three patients exhibited clinical BPH, whereas in the group with non-severe periodontitis, five patients exhibited clinical BPH. The prevalence of BPH and its associated indicators did not differ significantly between the severe and non-severe periodontitis groups. Our findings seemed to contradict with results from certain previous studies.

Numerous prior studies have demonstrated a correlation between periodontitis and BPH. For instance, an observational study conducted by Lan Wu et al. on a Chinese population revealed that the presence of periodontitis significantly elevated the risk of BPH (OR = 4.18) (Wu et al., 2019). Another observational study in a Korean population conducted by Hana Hyun et al. revealed that there was no statistically significant difference in the proportion of patients with PV ≥ 30 mL (*p* = 0.311) or IPSS ≥8 (*p* = 0.238) between the groups with and without periodontitis. However, according to the study’s diagnostic criteria for BPH (IPSS ≥8, maximal flow rate <5 mL/s, and PV ≥ 30 mL), there was a significant difference in BPH occurrence between the two groups (*p* = 0.037) (Hyun et al., 2021). Similarly, a study conducted on a Taiwanese population did not find a difference in the incidence of BPH A study based on a Taiwanese population similarly did not find a difference in BPH incidence between the groups with and without periodontitis (Fu et al., 2021). Our research results exhibit some similarities with the findings of Hana and Earl Fu, while contrasting with the findings of Lan Wu. We hypothesize that the dissimilarity in outcomes may stem from variations in the definitions of periodontitis and BPH across different studies. Drawing upon our clinical expertise, we have observed a relatively high prevalence of purely histologic BPH and mild periodontitis in middle-aged men, with limited clinical significance. Consequently, we adopted the diagnostic criteria of clinical BPH and severe periodontitis for comparative analysis, aligning with the most recent guidelines and relevant literature (Barry et al., 2017; Papapanou et al., 2018). The diagnostic criteria employed in Hana’s study exhibited similarities to our own, whereas Lan Wu’s study diverged by utilizing the community periodontal index rather than CAL for periodontitis diagnosis, resulting in a notable disparity. The dissimilarity in outcomes may also be attributed to the potential error stemming from the limited sample size. Consequently, we opted to conduct a more comprehensive analysis of the potential causal relationship between periodontitis and BPH through MR analysis, as it is less prone to confounding or reverse causality (Birney, 2022). Furthermore, the substantial sample size of the GWAS enhances the statistical power of our study. This study represents the inaugural attempt to assess the causal association between periodontitis and BPH through the utilization of two-sample MR analysis. All 5 MR analysis techniques, encompassing IVW method, consistently indicated the absence of a causal link between BPH and periodontitis. The finding was further corroborated by the reverse MR analysis. Furthermore, the absence of heterogeneity or pleiotropy was observed.

Current research has also explored non-genetic factors that heighten the likelihood of developing BPH in relation to periodontitis. The presence of ectopic oral flora may be a significant factor in the link between periodontitis and BPH (Alluri et al., 2021; Fang et al., 2021). The study has discovered the presence of a minimum of one oral pathogen, specifically porphyromonas gingivalis, which is a commonly identified causative agent of periodontitis, within the prostate secretions of patients who have both periodontitis and BPH (Estemalik et al., 2017). There is a proposed relationship between periodontitis and BPH mediated by gut flora (Guo et al., 2023). Additionally, the presence of specific proinflammatory cytokines originating from periodontal tissues potentially contributes to the development of systemic disorders such as BPH (Mazurek-Mochol et al., 2024). Our research indicates that periodontitis as a disease may not directly impact the occurrence of BPH; however, the dysbiosis of oral microbiota and alterations in the inflammatory state caused by periodontitis could potentially elevate the risk of developing BPH. Consequently, it would be prudent for individuals with periodontitis to closely monitor changes in their oral flora and inflammatory state.

The study’s primary strength lies in its integration of both observational study and MR analysis to assess the causal association between periodontitis and BPH, examining clinical and genetic perspectives, respectively. Cross-sectional studies provide an opportunity to investigate the association between periodontitis and BPH at an epidemiological level using self-reported data from a population sample. However, a limitation of cross-sectional studies is the inability to establish causality, leading to the formulation of only preliminary research hypotheses. Thus, in this study, we utilized MR analysis to infer a potential causal relationship between periodontitis and BPH following the initial findings from the cross-sectional study. MR analysis effectively mitigates the impacts of residual confusion, reverse causal deviation, and measurement errors commonly encountered in conventional epidemiological research. Furthermore, bidirectional analysis ensures the establishment of causal relationships between periodontitis and BPH in both directions. The integration of these two methodologies enhances the credibility and robustness of our findings compared to existing studies. Additionally, in the cross-sectional study, we employed more clinically significant criteria for diagnosing periodontitis and BPH compared to previous studies, enhancing the study’s potential to guie clinical treatment and prevent overtreatment. Although there are contradictory points between our results and some previous studies, our results offer a novel avenue for future research. Through the integration of observational studies and MR analysis, our findings can offer clinicians valuable insights to enhance their understanding of the relationship between periodontitis and BPH, ultimately improving clinical decision-making and treatment strategies.

Nevertheless, it is important to acknowledge the inherent limitations of this study. Specifically, the small sample size in the observational study may lead to potential errors, while the cross-sectional design could introduce recall bias on BPH diagnosis. Additionally, the exclusive use of European populations in the MR analysis may introduce regional bias and restrict the generalizability of the findings to the Chinese population. A larger sample size from diverse institutes is necessary to enhance the statistical power in future association studies. Additionally, conducting Mendelian randomization analysis using genetic data from Chinese populations can improve the accuracy of the conclusions.

The findings from our epidemiological investigation and MR analysis do not provide support for a causal relationship between periodontitis and BPH. More samples are needed to verify our results.

## Data Availability

The original contributions presented in the study are included in the article/Supplementary Material, further inquiries can be directed to the corresponding authors.
